# Voltage-induced Interface Reconstruction and Electrical Instability of the Ferromagnet-Semiconductor Device

**DOI:** 10.1038/s41598-017-00547-4

**Published:** 2017-03-23

**Authors:** Shu-Jui Chang, Po-Chun Chang, Wen-Chin Lin, Shao-Hua Lo, Liang-Chun Chang, Shang-Fan Lee, Yuan-Chieh Tseng

**Affiliations:** 1 Department of Materials Science & Engineering, National Chiao Tung University, Hsinchu, Taiwan; 2 Department of Physics, National Taiwan Normal University, Taipei, Taiwan; 3 Institute of Physics, Academia Sinica, Taipei, 30010 R.O.C. Taiwan

## Abstract

Using x-ray magnetic spectroscopy with *in-situ* electrical characterizations, we investigated the effects of external voltage on the spin-electronic and transport properties at the interface of a Fe/ZnO device. Layer-, element-, and spin-resolved information of the device was obtained by cross-tuning of the x-ray mode and photon energy, when voltage was applied. At the early stage of the operation, the device exhibited a low-resistance state featuring robust Fe-O bonds. However, the Fe-O bonds were broken with increasing voltage. Breaking of the Fe-O bonds caused the formation of oxygen vacancies and resulted in a high-resistance state. Such interface reconstruction was coupled to a charge-transfer effect via Fe-O hybridization, which suppressed/enhanced the magnetization/coercivity of Fe electronically. Nevertheless, the interface became stabilized with the metallic phase if the device was continuously polarized. During this stage, the spin-polarization of Fe was enhanced whereas the coercivity was lowered by voltage, but changes of both characteristics were reversible. This stage is desirable for spintronic device applications, owing to a different voltage-induced electronic transition compared to the first stage. The study enabled a straightforward detection of the spin-electronic state at the ferromagnet-semiconductor interface in relation to the transport and reversal properties during operation process of the device.

## Introduction

Ferromagnetic materials have a non-volatile nature at room temperature, but their magnetization can be switched reversibly with unlimited times. On the other hand, the electrical properties of semiconductors can be effectively altered by doping photons and electric fields, which provide amplification and transistor actions. Due to the impressive technological advantages of these two materials, their combination, called spintronics, emerges with an exploding technological impact. To make the control of magnetization fully compatible with current semiconductor devices, using external voltage has become a desirable means to control magnetization^1, 2^. Extensive studies have demonstrated that voltage can manipulate magnetic phase transformation^3, 4^, exchange bias^5, 6^, spin polarization^7–9^, and magnetic anisotropy^10–12^. These findings demonstrate the advance in spintronics because of the possibility of devices operating with reduced power consumption, and the compatibility of voltage-controlled devices with semiconductor integrated circuits. Although these findings have been recognized with great excitement, the underlying physics is an important yet unsolved problem, especially from the viewpoint of electronic interactions that regulate the magnetic ordering. A general explanation for the phenomena is the change in the occupation of ferromagnetic (FM) 3*d* orbital, due to the band-filling effect^10, 12, 13^. Despite many interpretations have been provided, evidence for the voltage-induced change in the spin-polarized electronic state is rarely reported due to experimental difficulty^14–18^. We hereby demonstrate the use of a technique on a ferromagnet/semiconductor device that is sensitive to the real-time change of the electronic state of FM with respect to the magnetic and transport properties of the device. By simultaneously operating x-ray magnetic spectroscopy and the voltage-control characterizations upon a Fe/ZnO device, we independently probed the behaviors of the ferromagnetic layer (Fe) out from the whole device during voltage application (including the spin electronic state, hysteresis, and anisotropy magneto-resistance). Such an element-resolved approach enabled a straightforward understanding of the phenomenon from the viewpoint of electronic states, thus making this work unique from previous studies that mainly approach the subject from a macroscopic setting. Of particular importance, the measurements were carried out in an ultra-high vacuum (UHV) condition (<10^−9^ Torr). Thus, we can rule out the possibility that the ambient condition may contribute to interfacial hybridization during voltage application. Hence, the observed modification of interfacial hybridization, along with the magnetic evolution, can be exclusively ascribed to the voltage effect alone. We observed, on a straightforward fashion, a Fe oxidation → metallic transition arising from 3*d* electronic redistribution with increasing voltage. The electronic transition resulted from a Fe-O charge-transfer effect is responsible for the change of Fe’s magnetization and coercivity (H_c_), both of which appeared reversible below a certain threshold voltage. Taking advantage of the helicity-dependent x-ray, the occupations of the majority- and minority-spin states of Fe, in conjunction with voltage-induced hybridization, were also explored. In an effort to rationalize and predict the behavior of voltage-controlled magnetism, this work delivers a close understanding of the local moments and electronic interactions from which this phenomenon is derived.

## Results and Discussions

**Figure 1:**
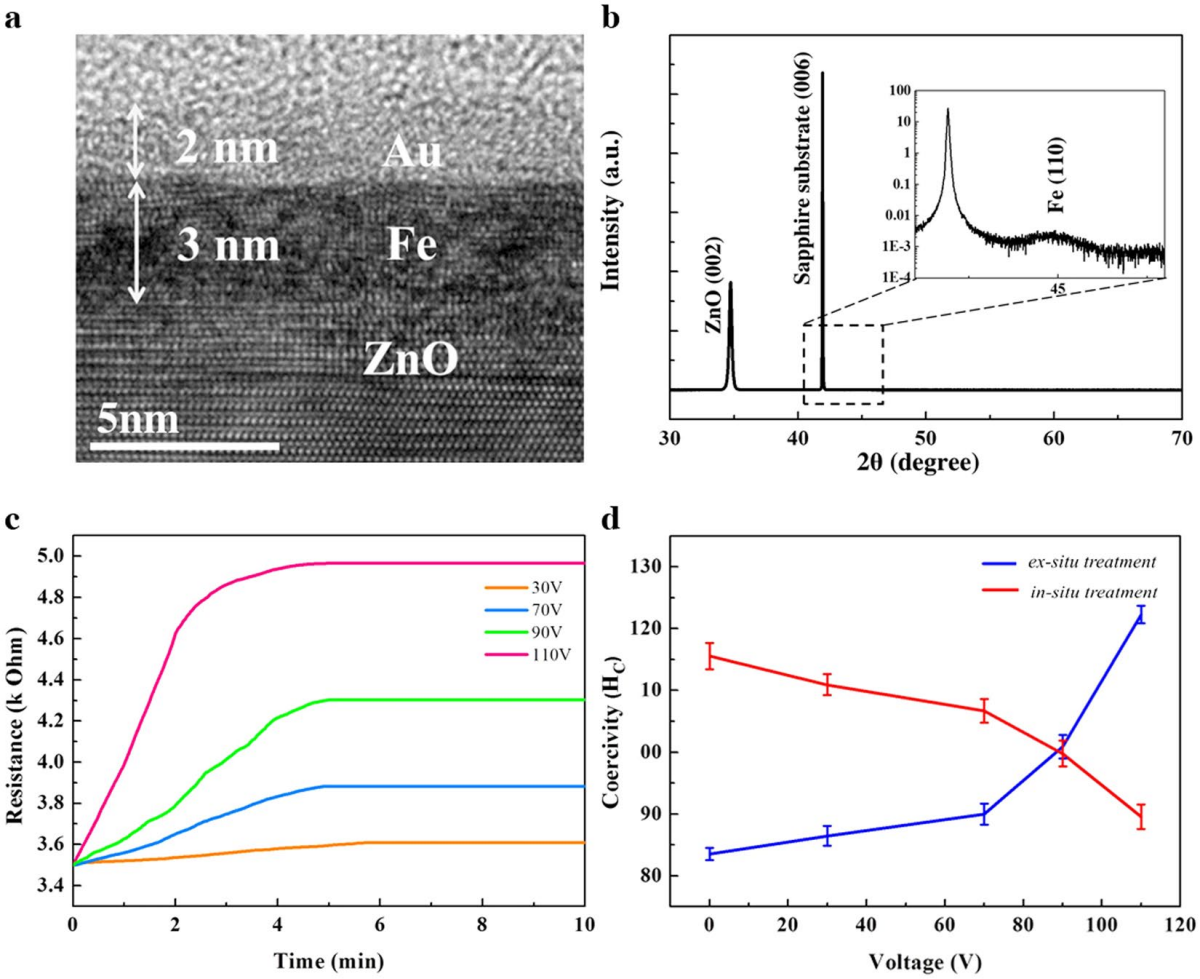
Structural, magnetic and electrical properties of the device. (**a**) Cross-sectional HRTEM imagine and (**b**) XRD pattern of the Au/Fe/ZnO hetero-structure device. (**c**) Time-dependent resistance of the device with 30, 70, 90, and 110 V treatments. (**d**) *Ex-situ*- and *in-situ treatment*-dependent H_c_ with varying voltages (MOKE). Inset of (**b**) shows zoom-in information of the Fe index.

Figure 1(a) shows a high resolution transmission electron microscopy (TEM) image of the Au (2 nm)/Fe (3 nm)/ZnO (300 nm) hetero-structure device. Figure 1(b) shows the x-ray diffraction (XRD) pattern of the sample with ZnO (002), Fe (110) and Al_2_O_3_ (006) indices. Figure 1(c) shows the time-dependent resistance of the device with varying applied voltages (30, 70, 90 and 110 V). Figure 1(c) shows the time-dependent current-perpendicular-to-plane (CPP) resistance of the device with varying applied voltages (30, 70, 90 and 110 V). The current densities of the sample for 30, 70, 90 and 110 V are 8.57 × 10^3^, 1.94 × 10^4^, 2.31 × 10^4^, and 2.55 × 10^4^ A/m^2^, respectively. The device appears electrically unstable for the first 5 min of operation during which the resistance gradually increases with time. This phenomenon is especially noticeable at 110 V. This implies that the electrical properties of the device have changed significantly at the beginning of operation, but turned stable afterwards. We find that the electrically unstable state is irreversible with the applied voltage. The resistance can never change back to the initial value even if the voltage is turned off. This implies a permanent change induced by the external voltage. We therefore performed two types of treatments on the device, *ex-situ* and *in-situ* voltage measurements (defined as *ex-situ* and *in-situ treatment* henceforth, respectively), to distinguish the two electrical states. The resultant magnetic properties were first examined by magneto-optic Kerr effect (MOKE). For *ex-situ treatment*, the voltage was applied on the device for 5 min, and data were collected after the removal of the applied voltage. *Ex-situ treatment* reflects the change of magnetic state of the device at the early stage of operation, during which the device underwent an irreversible, electrically unstable transition. In contrast, for *in-situ treatment*, data were collected after a 8-min operation, and the voltage was continuously supplied during data collection. *in-situ treatment* refers to the real-time change of magnetic state of the device for constant operation. During this process, the device entered into an electrically stable state. Figure 1(d) presents the *ex-situ* and *in-situ treatment* -MOKE results with voltage-dependency, where coercivity (H_c_) in *ex-situ treatment* is found to increase with the applied voltage. However, the *in-situ* treated sample displays an opposite voltage dependency in H_c_. The details of the *ex-situ*- and *in-situ treatment* - hysteresis loops are demonstrated in Fig. S1 (ESI). Apparently the two states correspond to two different magnetic behaviors in response to the applied voltage.

**Figure 2:**
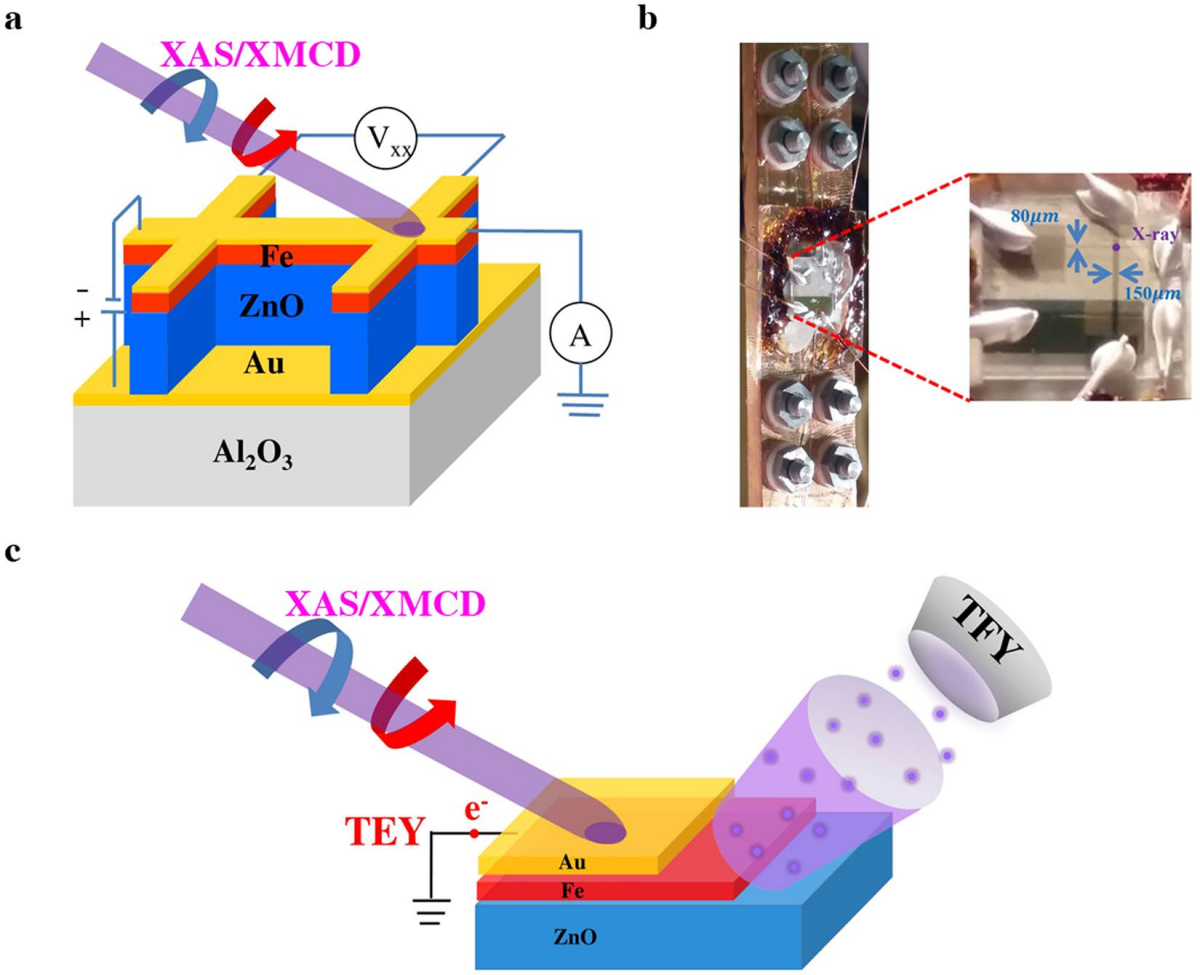
*In-situ* x-ray setup and deice layout. (**a**) Illustration for the geometric layout of the Hall bar device with respect to x-ray measurements (XAS/XMCD) under the applied voltage (polarity is shown). For x-ray measurements, voltage was applied along vertical direction of the device (CPP). While for AMR measurements, voltage was applied along horizontal direction of the device (CIP). (**b**) The real configuration of employed circuits on the device and the x-ray sample holder. (**c**) Illustration for the tuning of x-ray collection mode in relation to sample profiling (i.e., TEY and TFY are surface and bulk sensitive, respectively). The zoom-in figure of (**b**) shows the details (pattern linewidth, connection, and x-ray hitting spot) of the device layout on the x-ray holder.

We then focus on the x-ray characterizations with *in-situ* electrical measurements in order to explore the mechanism responsible for the observed phenomenon. Figure 2(a) illustrates the geometric layout of the Hall bar device with respect to x-ray measurements under the applied voltage. The voltage was applied along vertical direction of the device. The Hall bar device also allows anisotropy magneto-resistance (AMR) measurement along horizontal direction of the device. For this Hall bar device, six electrodes were placed in a slightly asymmetric manner for the ease of x-ray measurements (demonstrated in Fig. S2 (ESI). This design made it feasible for an x-ray to shine on the cross of the patterned lines while electrical measurement was performed on the device. It is noteworthy that all presented x-ray spectra were collected from the same sample spot of the device to ensure data consistency and accuracy. During x-ray measurements, the x-ray beam size was cut by a slit with a dimension close to the patterned linewidth. Figure 2(b) demonstrates the real configuration of the employed circuits on the device and the x-ray sample holder. The six electrodes were connected to the pins with Cu wires for electrical polarization. These pins were wounded by enameled wires with the other ends fixed by Mo screws located at the top of the holder. These Mo screws were independently connected to different cables going outside the x-ray chamber, so that they could be electrically controlled by a remote Hall measurement system. To ensure the accuracy, all the connecting wires were of equal length, in order to avoid possible variations in sheet resistance, carrier concentration, and electron mobility. The sample holder was then placed between a 0.5 Tesla electro-magnet inside a soft x-ray UHV chamber for spectroscopic measurements. Figure 2(c) illustrates the x-ray measurement modes in relation to sample profiling in this study. By tuning the x-ray mode (total electron yield (TEY) and total fluorescence (TFY) mode) and photon energy (Fe *L*_*3*_- and O *K*-edges), we were able to independently probe information from the Fe layer (Fe *L*_*2*_ */L*_*3*_-edge by TEY mode), Fe/ZnO interface (O *K*-edge by TEY mode), and ZnO bulk (O *K*-edge by TFY mode). The cross-tuning of x-ray mode and photon energy provides the most critical measure to explore the voltage-induced effects on different regions of the device, with element-specificity and spin-dependency.

**Figure 3:**
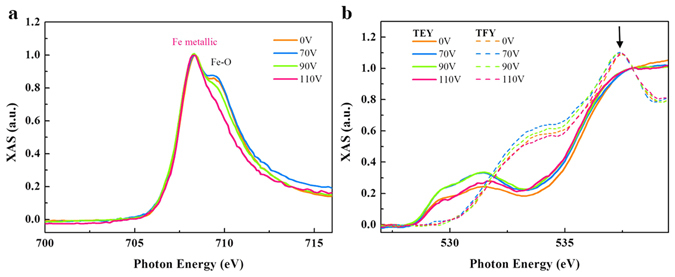
Voltage-dependent x-ray results. (**a**) Voltage-dependent Fe *L*_*3*_ XAS with *ex-situ treatment*, collected by TEY mode. XAS peaks corresponding to Fe-metallic and Fe-O features are marked. (**b**) Voltage dependent O *K*-edge XAS with *ex-situ treatment*, collected by TEY (solid lines) and TFY (dashed lines) modes. Arrow indicates Fe-O hybridization with an O termination of the ZnO layer adjacent to the Fe layer. All the XAS spectra have been normalized to the absorption jump.

Figure 3(a) presents the Fe *L*_*3*_ x-ray absorption spectroscopy (XAS) spectra with *ex-situ treatment* of 0, 70, 90, and 110 V (30 V is not presented for the clarity of the figure). Without the applied voltage, Fe *L*_*3*_ XAS displays a shoulder-like feature at photon energy of ~710 eV. This feature is a clear indicator to the oxidation state of the Fe layer that arose from Fe 3*d*-O 2*p* hybridization with ZnO. However, the characteristic is suppressed with increasing voltage, and finally disappears at 110 V. This suggests that Fe undergoes an oxidized → metallic transition with increasing *ex-situ treatment*; i.e., the external voltage breaks the Fe-O bond. This bond-breaking action reconstructs the interface and is therefore responsible for the increase in resistance, which is an irreversible change. Figure 3(b) shows TEY O *K*-edge XAS with the same *ex-situ treatments*. The data reflect the Fe/ZnO interface information. The oscillating nature of the spectra originates from Fe 3*d*-O 2*p* hybridization that results in the splitting of Fe-3*d* states by the ligand field. However, when turning the x-ray mode to TFY, the O spectral line-shape redistributes, which is superimposed in Fig. 3(b) (dashed lines). The reformed XAS is associated with the O 2*p*-Zn 4 *s* hybridization originating from the ZnO bulk instead of from the interface. The significant shoulder observed at 537 eV (indicated by arrow) indicates an O termination of the ZnO layer adjacent to the Fe layer^19, 20^. The O termination is responsible for the oxidation state of the Fe layer without the applied voltage, in agreement with the O *K*-edge TEY results. Of particular importance, both TEY and TFY O *K*-edge XAS show voltage dependency. Since the XAS intensity is proportional to the number of unoccupied states, the voltage-dependent Fe *L*_*3*_ */*O *K*-edge XAS suggests a charge-transfer effect taking place within the device. The charge transfer is the underlying physics behind interface reconstruction. It is voltage-driven, and overwhelmingly dominates the electronic properties of the device, as it electronically modifies the FM layer, FM/semiconductor interface, and semiconductor bulk.

**Figure 4:**
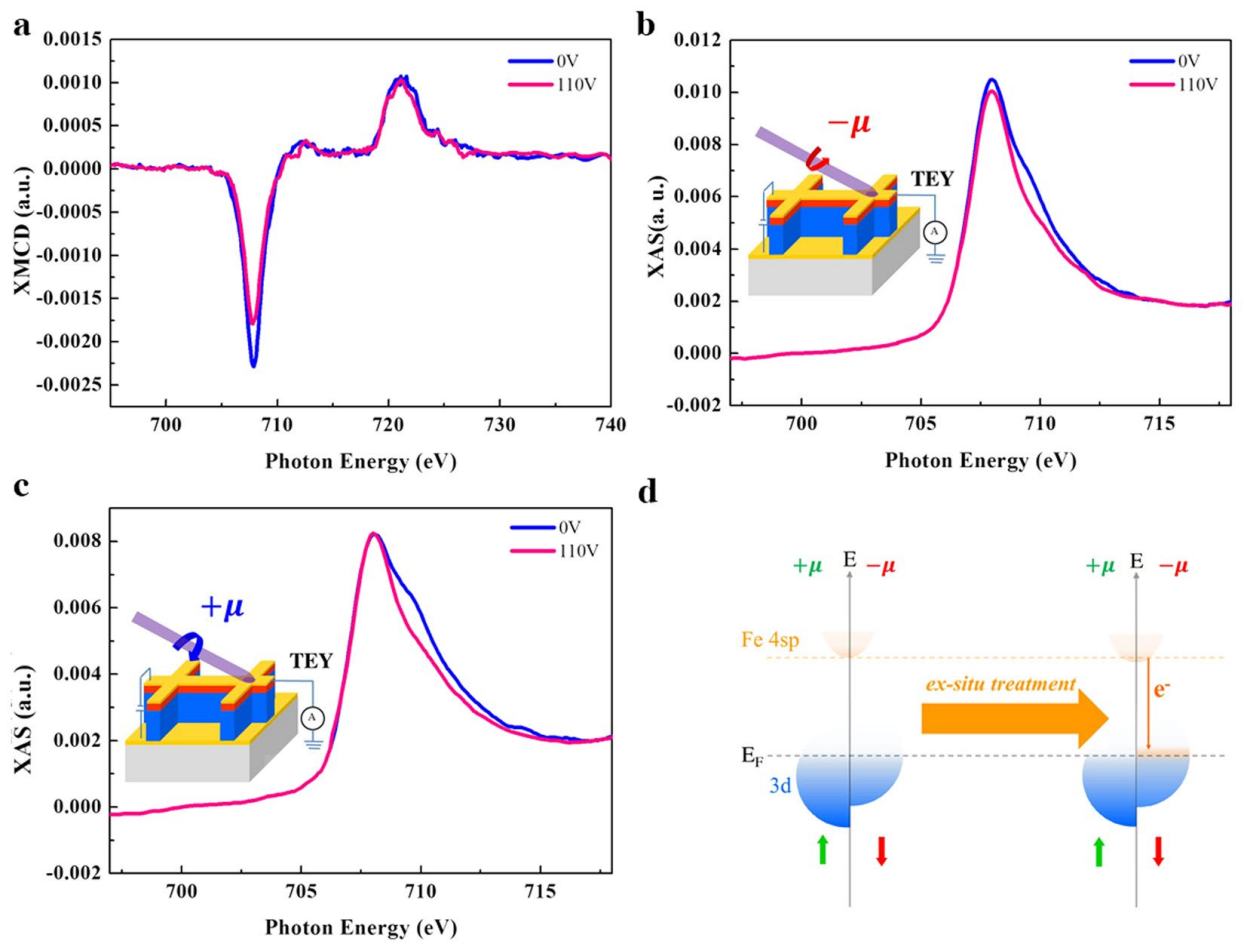
Helicity/spin-resolved x-ray results. (**a**) Fe *L*_*2*_/*L*_*3*_ XMCD spectra with and without 110 V- *ex-situ treatment*. (**b**) Helicity-dependent (μ(+) and μ(−)) Fe *L*_*3*_ XAS spectra with and without 110 V-*ex-situ treatment*. Inset of (**b**) illustrates the x-ray measurements with two x-ray helicities. (**d**) Illustration for the spin-dependent electronic diagram of the Fe layer in relation to the charge transfer effect upon *ex-situ treatment*. μ(+) and μ(−) correspond to the different x-ray helicities associated with majority and minority state, respectively. The charge transfer only involves the change in Fe’s minority state.

We find that the charge transfer is accompanied by the change of spin-electronic states of the Fe layer. Figure 4(a) provides the x-ray magnetic circular dichroism (XMCD) spectra before and after the 110-V *ex-situ treatment*. The XMCD signal appears to decrease upon voltage treatment. This is indicative of the suppression of Fe atomic moment. We collected helicity-dependent Fe *L*_*3*_-edge XAS for both 0- and 110-V *ex-situ treatments*. We define μ(+) and μ(−) as the XAS spectra with positive and negative helicities generated from circularly polarized x-rays, respectively. Therefore, μ(+) and μ(−) intensities are inversely proportional to the occupations of majority- and minority-spin states of Fe 3*d* orbital, respectively. The minority spin states (as shown by μ(−) intensity in Fig. 4(b)) of Fe appear to decrease upon *ex-situ treatment*. However, the majority spin states (as shown by μ(+) intensity in Fig. 4(c)) are independent of the applied voltage (the shoulder feature of 110 V- *ex-situ treatment* μ(+) around 710 eV is associated with the oxidation → metallic transition, while this feature is independent of magnetic as it appears in both with and without 110 V- *ex-situ treatment* μ(+) XAS). The discrepancy between minority and majority states of Fe, therefore, is the origin of the change of Fe atomic moment in response to interface reconstruction. We correlate this with TEY O *K*-edge XAS (Fig. 3(b)) which provides information of Fe-O hybridization at the Fe/ZnO interface. The pre-peaks of TEY O *K*-edge XAS are split by octahedral ligand-fields with t_2g_ and e_g_ states for the 3*d* metals. Upon *ex-situ treatment*, the double-peak feature turns to a single-peak feature at ~530 eV, whereas the major peak (marked as E) at ~537 eV appears more prominent. This is a clear evidence for the change of Fe 4*sp*-O 2*p* hybridization^21–23^, and this electronic transition is responsible for the decrease of the Fe atomic moment by interface reconstruction. Moreover, when correlating with helicity (spin)-dependent Fe *L*_*3*_-edge XAS, we find that the Fe-O electronic transition involves the change of minority states via the charge-transfer mechanism; i.e., electrons only follow from the ZnO to Fe’s minority state through Fe 4*sp*-O 2*p* hybridization. The spin-dependent electronic diagram of the Fe layer, with respect to interface reconstruction (i. e., *ex-situ treatment*), is summarized in Fig. 4(d). The spin polarization of the Fe layer is only influenced by the minority spin state. Upon interface reconstruction, the occupation of the Fe minority state increases due to the charge donated by the ZnO. This leads to a decrease of the Fe spin polarization as the band-splitting is reduced. We notice that the charge transfer suppressed the Fe magnetization mainly by lowering its orbital moment (*L*_*z*_). This is implied by the noticeable changes of both *L*_*3*_-edge XAS and XMCD spectrum, whereas *L*_*2*_ XAS and XMCD remain nearly unchanged with the applied voltage. According to the XMCD sum rules^24^, the *L*_*3*_-edge is corresponsive to *L*_*z*_ change, whereas the *L*_*2*_-edge is responsible for *S*_*z*_ variation for 3d metals. It is well known that *L*_*z*_ is far more sensitive than *S*_*z*_ to structural transition. Unlike *S*_*z*_, which can freely switch upon field reversal, *L*_*z*_ is often bound to lattice and structural coordination. Therefore, the voltage-induced interface reconstruction is expected to result in broken-symmetry effects reflected by *L*_*z*_ modification.

**Figure 5:**
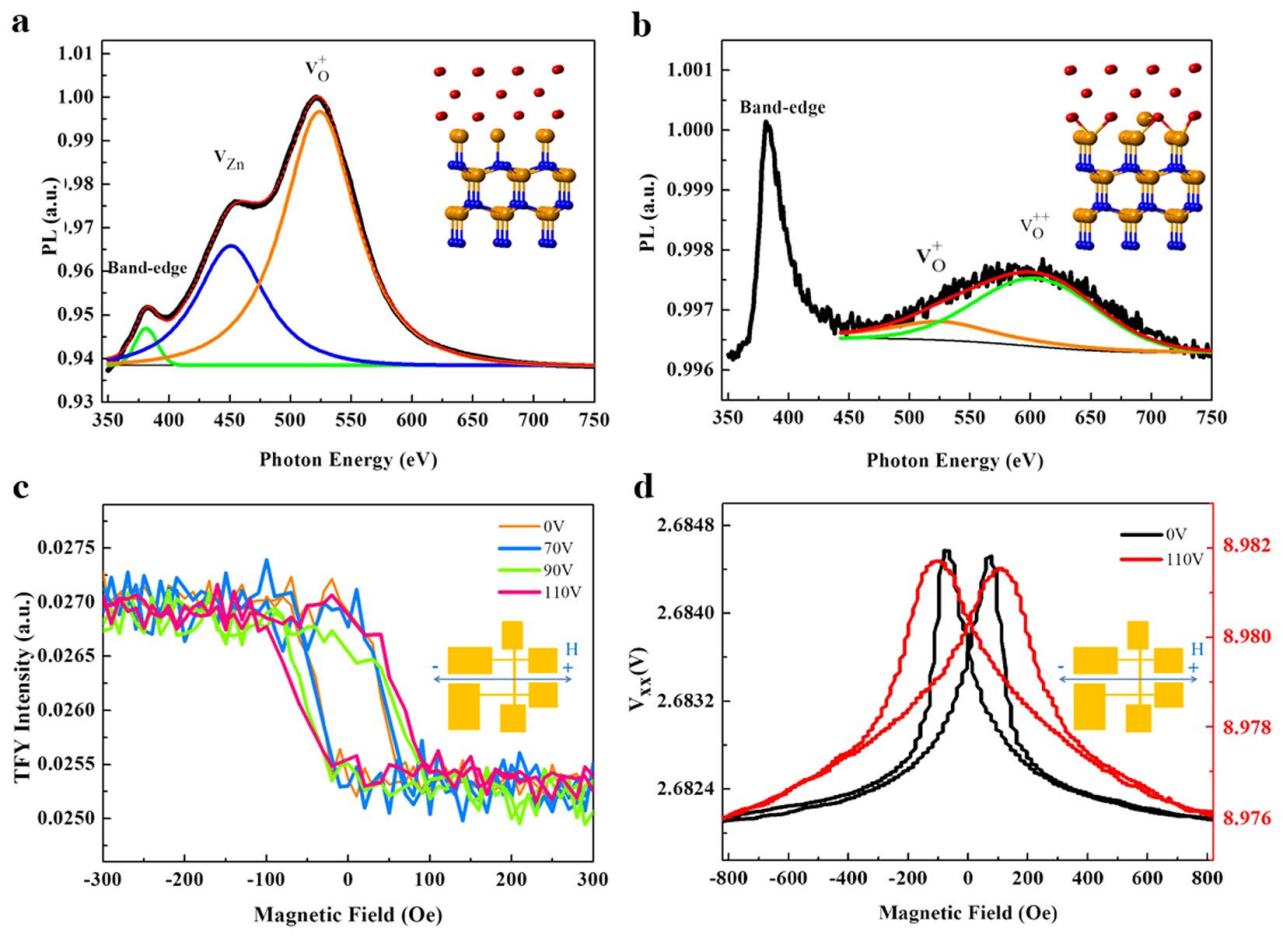
Magnetic properties with *ex-situ treatment*. PL spectra for the device (**a**) with and (**b**) without 110 V- *ex-situ treatment*. Inset figures of (**a**) and (**b**) illustrate the Fe-ZnO bond-broken and bond-formed situations for with and without 110 V-*ex-situ treatment*, respectively. (**c**) Voltage-dependent hysteresis curves of the Fe layer with *ex-situ treatment*. (**d**) AMR measurement of the device with (red curve, units on right y-axis) and without (black curve, units on left y-axis) 110 V-*ex-situ treatment*. Magnetic field direction with respect to the patterned sample is shown in inset figures.

We then take a further look at the correlation between charge transfer and interface reconstruction. We took photoluminescence (PL) spectra for the samples with and without 110-V *ex-situ treatment*, as shown in Fig. 5(a) and (b), respectively. Without *ex-situ treatment*, the band edge emission at 380 eV is attributed to free excitonic emission of ZnO, whereas the broad peak is attributed to extrinsic defect sites due to the recombination of photo-generated holes with electrons occupying the singly ionized oxygen vacancies (O_v_)^25–28^. Upon *ex-situ treatment*, O_v_ becomes the dominant characteristic. O_v_ must be present at the Fe/ZnO interface that caused Fe-O bond-breaking. One can imagine that O_v_ serve as a hub for electrons that are readily available to follow from ZnO to Fe when an electric field (i.e., the applied voltage) is given. The formation of O_v_ also explains the increase in resistance by interface reconstruction. We then take the element-specific hysteresis curves on the Fe layer by fixing photon energy at the Fe *L*_*3*_-edge upon the magnetic field reversal. Figure 5(c) shows the voltage-dependent hysteresis curves of the Fe layer. We observe a voltage-induced increase of switching field with increasing *ex-situ treatment*. Independently we also collected AMR data of *ex-situ treatment* −110 V (Fig. 5(d)). The increase of switching field in AMR is consistent with H_c_ enhancement. From the viewpoint of magnetic reversal, O_v_ could act as domain-wall pinning centers. As shown in many previous studies^29, 30^ it is well known that crystal defects tend to pin the domain wall during magnetic reversal. This caused increase in both H_c_ and AMR switching field.

**Figure 6:**
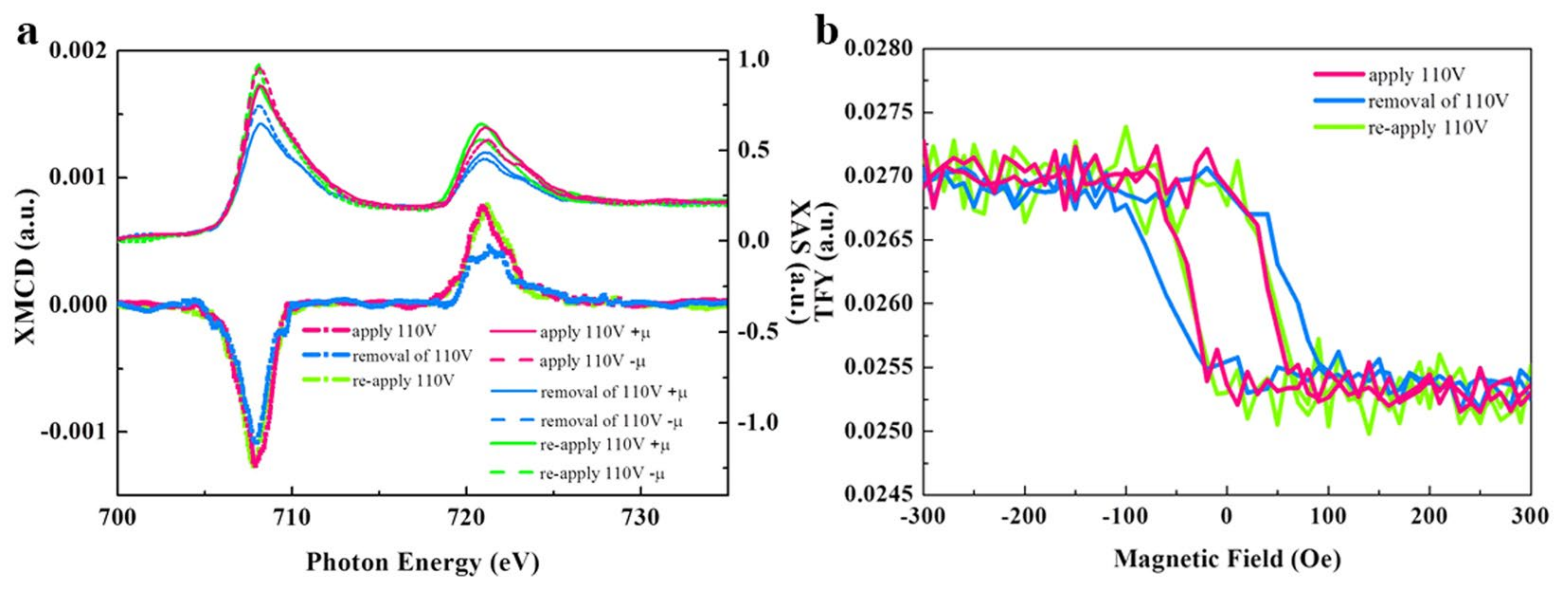
Reversibility test of *in-situ* treatment by x-ray. (**a**) Lower figure: Fe *L*_*2*_/*L*_*3*_ XMCD spectra with application (pink), removal (blue), and reapplication (green) of 110 V-*in-situ treatment*; upper figure: helicity-dependent (μ(+) and μ(−)) Fe *L*_*3*_ XAS spectra with and without 110 V- *in-situ treatment*. (**b**) Hysteresis curves of the Fe layer with application (pink), removal (blue), and reapplication (green) of 110 V- *in-situ treatment*.

Next, we discuss the *in-situ treatment* results. Lower figure of Fig. 6(a) shows the results of Fe *L*_*2*_ */L*_*3*_-edge XMCD for a prolonged supply of a 110-V on the device (>8 minutes), followed by a temporary removal of the voltage, and then a re-application of the 110-V on the device. For *in-situ treatment*, the device has passed the interface reconstruction period and entered into an electrically stable state. Contrary to *ex-situ treatment*, the XMCD signal is enhanced with *in-situ treatment*. We find that the XMCD returns to original magnitude upon the removal of *in-situ treatment*, and it can be re-enhanced by re-applying *in-situ treatment*. This means a reversible control of the transition with the applied voltage. As shown by helicity-dependent XAS (upper figure of Fig. 6(a)), we find that the moment increase involves the change of both majority and minority states. Besides, both the *L*_*2*_-edge (*L*_*z*_-related) and *L*_*3*_-edge (*S*_*z*_-related) XMCD signals are promoted with the applied voltage, which implies an intrinsic increase of the Fe atomic moment. This indicates a distinct voltage-induced electronic transition compared to the *ex-situ treatment*, because of the completion of the interface reconstruction. For this type of electronic transition, the spin-polarization (i.e., XMCD) of the FM layer is enhanced, due to the change of both majority and minority states by electronic filling. Interestingly, the enhanced spin-polarization is accompanied by the decrease of H_c_ (Fig. 6(b)) in which the reversibility is also observed. We find that 110 V is the maximum voltage that can allow the reversible behavior. The spin-electronic transition will become permanent if the voltage exceeds this value. This indicates the operation limit of the device.

## Conclusion

In summary, we have extensively investigated the voltage effects on the Fe/ZnO device by directly probing the Fe/ZnO spin-electronic state, with a strong attempt to capture the spirit of the voltage-controlled magnetism that has been emerging in recent years. Our results are important in several aspects. First, the Fe/ZnO interface was reconstructed by the applied voltage at the early stage of device operation. In this stage, the resistance was raised up due to Fe-O bond breaking, and O_v_ was formed at the interface. With a continuous supply of voltage, the metallic phase of the Fe layer was stabilized, thus turning the device into an electrically stable state. In this state, the external voltage drove a different electronic transition from the first state, in which the spin-polarization was enhanced along with a reduction of the switching field. In this state we found that both characteristics were reversible, as long as the applied voltage was kept below a threshold value. This finding offers an optimistic view for the FM/SM design: (i) the spin-polarization enhancement suggests a probable increase of spin current that could be injected into the semiconductor; (ii) the decrease of H_c_ infers a lower switching current for device operation. Both characteristics are desirable for spintronics applications. However, there is a serious concern about how to remove the negative impacts of interface reconstruction that occur at the early operation stage (i.e., causing electrically unstable state and H_c_ increase). This stage causes considerable power-consumption for the device as a result of the increase in resistance and lowering in switching speed. This problem might be solved by having a Zn-rich, instead of O-rich surface prior to depositing the Fe layer, or inserting a non-magnetic, highly conductive interlayer between the Fe and ZnO. Second, ZnO is a good candidate for spin pumping owing to its Rashba-type spin-orbital interaction that may give rise to a long-distance spin transport^31, 32^. If the interface-reconstruction effect could be mitigated to some extent, a longer spin-relaxation time might be achieved for ZnO in combination with 3*d* metals. This will allow a better control of the spin electrons in ZnO. This work is supported by the Ministry of Science & Technology, Taiwan, under Grant No. MOST 104-2633-M-009-001.

## Methods

### Device fabrication

Multi-layers of Au/Fe/ZnO/Au were sequentially deposited on an Al_2_O_3_(0001) substrate. The Au and Fe layers were deposited using e-beam-heated evaporators while the ZnO thin films were prepared using a pulsed laser deposition technique^33, 34^. A Hall bar structure of Au/Fe/ZnO/Au/Al_2_O_3_ was fabricated using the lithography technique for transport measurement.

### Structural characterizations

XRD was performed at BL17B1, National Synchrotron Radiation Research Center (NSRRC), Taiwan, with a photon energy of 8 keV. TEM was used to examine the interface microstructure.

### Magnetic and photoluminescence measurements

Magnetic properties (hysteresis curves) of the device were measured using a MOKE system, equipped with an electromagnet that provides a magnetic field range of ±1000 Oe. PL spectra were collected using a Kimmon IK3001R-G system equipped with a He-Cd laser (720 W).

### X-ray magnetic spectroscopy and transport measurements

X-ray magnetic spectroscopy was operated at BL11A (Dragon beamline, NSRRC). This beamline was incorporated with an electrical control system allowing simultaneous measurements of XAS, XMCD, and electrical transport. More details about the setup can be found in ref. 35. Resistance in the CPP geometry and element-specific hysteresis curve measurements were operated on the Fe layer by tuning the photon energy to the Fe *L*_*3*_ absorption edge upon the magnetic field reversal at the same beamline. An independent four-point probe AMR measurement in the current-in-plane geometry and magnetization hysteresis loop were performed along horizontal direction of the device, using a magnetoresistance (MR) and a longitudinal micro-MOKE technique^36^. The detection of x-ray absorption spectra was taken via a TEY mode and a TFY mode to probe the surface- and bulk-sensitive information of the device, all in an UHV condition (<10^−9^ Torr). Two voltage treatments were carried out in this study, as described in the content of results and discussion. A sample CPP resistance of 3.5 kohm was independently measured prior to the x-ray measurement. The same sample resistance was obtained by the electrical control system incorporated to the x-ray setup. During measurements, the sample was maintained at 200 K using a temperature controller in order to prevent voltage-induced heating and thermal gradient on the device. Device breakdown occurred at 130 V, at which TEY/TFY signal cannot be obtained anymore. Therefore, the maximum applied voltage to the device was set to be 110 V.

## Electronic supplementary material

Supporting information
