# Supplementary material for: Voltage-induced Interface Reconstruction and Electrical Instability of the Ferromagnet-Semiconductor Device
Source: Sci Rep. 2017 Mar 23;7:339. doi: 10.1038/s41598-017-00547-4 (PMC5428722; doi:10.1038/s41598-017-00547-4)
Supplement: Supplementary file 1 — Supporting information [file 41598_2017_547_MOESM1_ESM.pdf]

Supporting information

Voltage-induced Interface Reconstruction and Electrical Instability of the Ferromagnet-Semiconductor Device

Shu-Jui Chang[1], Po-Chun Chang[2], Wen-Chin Lin[2], Shao-Hua Lo[1], Liang-Chun Chang[3], Shang-Fang Lee[3], and Yuan-Chieh Tseng[1*]

1. Dept. Materials Science & Engineering, National Chiao Tung University, Taiwan.

2. Dept. Physics, National Taiwan Normal University, Taiwan.

3. Institute of Physics, Academia Sinica, Taiwan

E-mail address:yctseng21@mail.nctu.edu.tw.

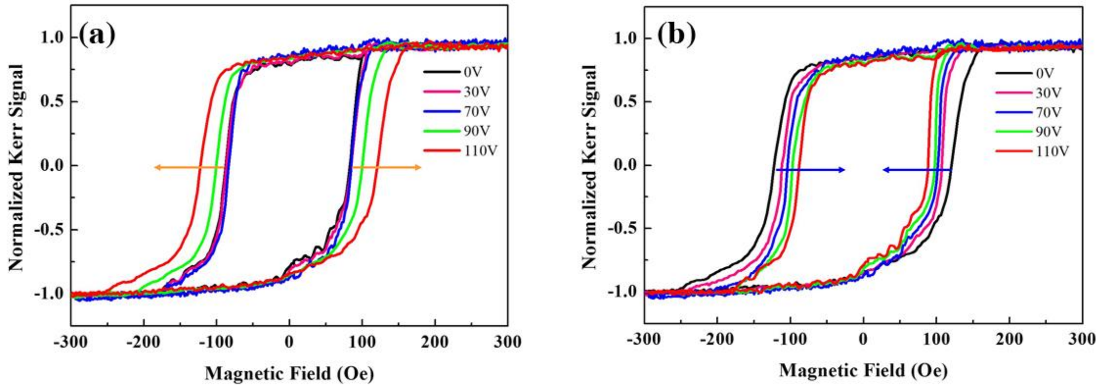

Fig. S1 The voltage-dependent MOKE hysteresis loops for sample with (a) *ex-situ*- and (b) *in-situ-treatments*. The coercivity is found to increase with increasing applied voltage with ESV treatment, whereas it decreases with increasing applied voltage with ISV treatment.

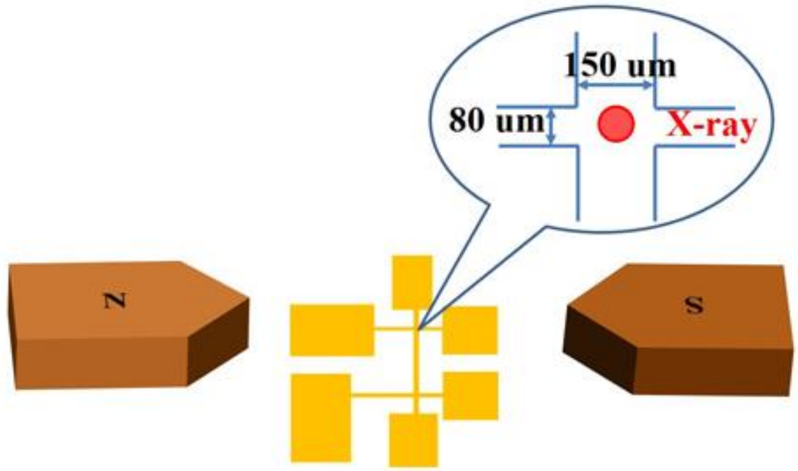

Fig. S2 the Hall bar design mounted on the E-X setup holder with respect to the electromagnet, in which a magnetic field up to 0.5 Tesla is applied along the horizontal (in-plane) direction of the film. The Hall-bar pattern is with an asymmetric geometry for the sake of easily finding the sample spot (red spot) during the x-ray measurements. The pattern dimension (~150μm × 80μm) relative to x-ray spot size (~80μm × 80μm) is shown in the zoom-in figure.
